# Association of vitamin D receptor gene polymorphisms with gestational diabetes mellitus-a case control study in Wuhan, China

**DOI:** 10.1186/s12884-021-03621-y

**Published:** 2021-02-17

**Authors:** Jianqiong Liu, Qiong Dai, Wei Li, Yan Guo, Anna Dai, Yanqing Wang, Mengyao Deng, Zhao Tang, Lu She, Xiaohong Chen, Mei Yang

**Affiliations:** 1 Maternal and Child Health Hospital of Hubei Province, Tongji Medical College, Huazhong University of Science and Technology, No.745 Wuluo Road, Wuhan, China; 2 Department of non-communicable chronic disease, Wuhan Centers for Disease Prevention and Control, Wuhan, China; 3 School of Basic Medicine, Tongji Medical College, Huazhong University of Science and Technology, Wuhan, China; 4 School of Medicine, Wuhan University of Science and Technology, No.947 Heping Road, Wuhan, China; 5 Research Center for Health Promotion in Women, Youth and Children, Wuhan University of Science and Technology, Wuhan, China

**Keywords:** Gestational diabetes mellitus, Vitamin D receptor, Single nucleotide polymorphisms, Multifactor dimensionality reduction, Gene‐gene interactions

## Abstract

**Background:**

Gestational diabetes mellitus (GDM) increased risk of perinatal complications for both the women and the fetuses. The association between the vitamin D receptor (VDR) gene polymorphism and GDM has not been thoroughly investigated in Chinese pregnant women. Therefore, we aimed to determine whether VDR gene single nucleotide polymorphisms (SNPs) rs154410, rs7975232, rs731236, rs2228570 and rs739837 contribute to GDM risk in Wuhan, China. Moreover, we aimed to explore their combined effects on the risk of GDM.

**Methods:**

Pregnant women who had prenatal examinations at 24 to 28 weeks’ gestation in our hospital from January 15, 2018 to March 31, 2019 were included in this case-control study. After exclusion, a total of 1684 pregnant women (826 GDM patients and 858 non-diabetic controls) were recruited. The clinical information and blood samples were collected by trained interviewers and nurses. Genotyping of candidate SNPs was conducted on the Sequenom MassARRAY platform. Statistical analyses including t-test, ANOVA, chi-square test and logistic regression were performed to the data with SPSS Software to evaluate differences in genotype distribution and associations with GDM risk. Multifactor dimensionality reduction method was used to explore the gene-gene interactions on the risk of GDM.

**Results:**

Differences in age, pre-pregnancy BMI, family history of diabetes and previous history of GDM between the case and control groups were statistically significant (*P* < 0.05), whereas no significant differences were found in height, gravidity, parity, and age of menarche (*P >* 0.05). There were no significant differences at genotype distributions of the examined VDR gene SNPs (*P >* 0.05). After adjusting by age, pre-pregnancy BMI, family history of diabetes, the results of logistic regression analysis showed no associations of the five SNPs with GDM in all the four genotype models(*P >* 0.05). Furthermore, there were no gene-gene interactions on the GDM risk among the five examined VDR gene SNPs.

**Conclusions:**

The VDR gene SNPs rs154410, rs7975232, rs731236, rs2228570 and rs739837 showed neither significant associations nor gene-gene interactions with GDM in Wuhan, China.

## Background

Gestational diabetes mellitus (GDM) is defined as glucose intolerance with onset or first recognition during pregnancy [1]. The pooled prevalence of GDM which ranges from 5.4 to 14.8 % depending on the populations is increasing worldwide [2–5]. The most frequent perinatal complications of GDM are macrosomia, primary cesarean delivery, clinical neonatal hypoglycemia, fetal hyperinsulinemia, premature delivery, shoulder dystocia or birth injury, need for intensive neonatal care, hyperbilirubinemia, and preeclampsia [6, 7]. In severe cases, GDM can lead to prenatal death. Therefore, it is essential to identify potential risk factors of GDM for the health of women and children.

Although its exact etiology is unknown, genetic variations related to ß-cell dysfunction and insulin resistance have been shown to contribute to the development of GDM [8, 9]. Given the fact that women with a history of GDM are at an increased risk of developing type 2 diabetes (T2D) later in their lives [10] and women with a family history of diabetes may be predisposed to an increased risk of GDM [11], it is plausible to hypothesize that GDM may share the similar risk factors and genetic susceptibilities with T2D [12].

The vitamin D receptor (VDR) is a member of the large family of nuclear receptor transcription factors and specifically binds the micronutrient-derived hormone 1α,25(OH)_2_D_3_ [13]. The role for this receptor in T2D has been widely studied in recent years [14–17]. These findings have generated considerable interest in the association of VDR and GDM [18–22]. However, the conclusions were conflicting and the confounding factors and interactions between genetic polymorphisms were commonly neglected. Moreover, most analyses were limited by only examining one or two single nucleotide polymorphisms (SNPs). Therefore, according to genome-wide association studies of T2D, five SNPs rs154410, rs7975232, rs731236, rs2228570 and rs739837 were determined in the present case-control study along with their combined effects on the risk of GDM in Wuhan, China.

## Methods

### Study population

Pregnant women who had prenatal examination at the Obstetrics and Gynecology Clinic of Maternal and Child Health Hospital of Hubei Province from January 15, 2018 to March 31, 2019 were consecutively recruited. A two-hour, 75 g oral glucose tolerance test (OGTT) was performed for all pregnant women at 24 to 28 weeks’ gestation, which was assessed from the date of the last menstrual period. The diagnosis of GDM was based upon the criteria of International Association of Diabetes and Pregnancy Study Groups (IADPSG): a fasting glucose ≥ 5.1 mmol/L (92 mg/dl), or a one-hour result of ≥ 10.0 mmol/L (180 mg/dl), or a two-hour result of ≥ 8.5 mmol/L (153 mg/dl) [7]. Pregnant women who reached these thresholds were included in GDM group. The non-diabetic controls were randomly selected at the same outpatient clinic matched with testing date and gestation week. Exclusion criteria were: age < 18 years; pre-gestational diabetes; multiple pregnancies; pregnancies complicated with endocrine diseases such as polycystic ovary syndrome; any other medical condition that might affect glucose regulation; unable or unwilling to cooperate with the study. After exclusion, a total of 1684 pregnant women (826 GDM patients and 858 non-diabetic controls) were recruited in the study. All the subjects were unrelated Han Chinese and lived in Wuhan of Hubei Province, a central area of China.

### Data Collection

A standard questionnaire was used by the trained interviewers to obtain information from all subjects regarding age, family history of diabetes, pregnant condition and other medical issues. Measurements of body weight and height were made for all subjects and body mass index (BMI) was calculated based on these measurements. Pre-pregnancy weight was obtained through medical records. The methods were carried out in accordance with the principles of the Declaration of Helsinki.

### Selection and genotyping of SNPs

By tracking the literature, combined with genome-wide association studies of T2D and minor allele frequency (MAF) > 0.05 reported in Chinese population, we selected five SNPs that were commonly investigated on the risk of GDM for assessment. These SNPs were rs1544410, rs731236, rs7975232, rs2228570 and rs739837. At recruitment, maternal blood samples in the fasted state (8 to 12 h fast, no more than 12 h) were collected by skilled nurses. After that, 2 ml blood were immediately placed on ice and separated into plasma and cells within 30 min, then distributed in aliquots and stored at -80°C until analysis. Genomic DNA was isolated from 0.5 ml blood cells using the approved guideline of the Relax Gene Blood DNA System DP348 (Tiangen, China). Genotyping of candidate SNPs was conducted on the Sequenom MassARRAY platform (Sequenom Inc., San Diego, CA, USA). For quality control, 5 % of duplicate samples were independently reanalyzed in a blinded manner. The call rates of rs1544410, rs731236, rs7975232, rs2228570, rs739837 were respectively 98.93 %, 98.93 %, 98.81 %, 98.69 %, 98.99 %, which were all higher than the quality control standard (95 %).

### Statistical analysis

Normality of distribution for continuous variables was tested by the Kolmogorov-Smirnov test. Normal distribution data were presented as mean ± standard deviation (SD), and the differences among groups were compared by unpaired Student’s t-test or analysis of variance (ANOVA). For non-normal distribution data, the chi-square test was performed for comparison among groups. The differences in genotype distribution as well as consistency of genotype distribution with Hardy-Weinberg equilibrium (HWE) were tested by using the Chi-square test. The HWE was a principle stating that the genetic variation in a population will remain constant from one generation to the next in the absence of disturbing factors. Chi-square test of goodness of fit was used to measure the coincidence between the observed number of genotypes and the HWE of all genotype frequencies at the locus. If *P* was above 0.05, the sample of this genotype conformed to the law of genetic equilibrium, which suggested that the sample had good population representation. Logistic regression was performed to evaluate the association of the genotypes and GDM risk. All *P* were two-sided and if below 0.05 the results were considered statistically significant. Analyses were conducted using SPSS Software, Version 24.0 for Windows (SPSS Inc., Chicago, IL, USA). Multifactor dimensionality reduction (MDR) [23] method was used to explore the gene-gene interactions on the risk of GDM.

## Results

### Clinical characteristics of subjects

The clinical characteristics of the subjects were given in Table 1. The average age of GDM group and control group was 30.99 ± 4.57 and 28.85 ± 4.23 years, respectively. Differences in age, pre-pregnancy BMI, family history of diabetes and previous history of GDM between the case and control groups were statistically significant (*P <* 0.05). The GDM patients had higher levels of age and pre-pregnancy BMI than the controls. No significant differences were found in height, gravidity, parity, and age of menarche between the GDM patients and controls (*P >* 0.05).

**Table 1:** Clinical characteristics of the subjects

	GDM(*n* = 826)	Non-GDM (*n* = 858)	*P*
Age ^a^ (year)	30.99 ± 4.57	28.85 ± 4.23	**< 0.01**
Height (cm)	159.78 ± 4.71	159.52 ± 6.07	0.37
Pre-pregnancy BMI (kg/m^2^)	22.23 ± 3.74	20.89 ± 6.60	**< 0.01**
Family history of diabetes	246(29.90)	102(12.10)	**< 0.01**
Gravidity			0.05
1	289(35.90)	338(40.10)	
2	232(28.90)	254(30.10)	
≥ 3	283(35.20)	251(29.80)	
Parity			0.29
Multiparous	340(41.30)	332(38.70)	
previous history of GDM	47(5.69)	4(0.47)	**< 0.01**
Age of menarche ^b^ (year)	13.40 ± 1.40	13.34 ± 1.31	0.34

Data were given as the mean ± SD or as n (%), with the significance of differences between groups evaluated using t-test or χ^2^ test

^a^Age refers to the age at which the participant was enrolled in the study

^b^Age of menarche refers to the age at which the first menstruation took place

Abbreviations: *BMI* body mass index; *GDM* gestational diabetes mellitus; *SD* standard deviation

Bold represents significant *P*

### Association between VDR gene SNPs and GDM

The distributions of five VDR gene SNPs in the control group were all in HWE (*P >* 0.05). The genotype distribution of VDR gene SNPs and associations of these candidate SNPs with GDM were shown in Table 2. There were no significant differences at genotype distributions of the five VDR gene SNPs (*P >* 0.05). Moreover, the associations of these candidate SNPs with GDM were not significant in different genotype models between cases and controls (*P >* 0.05). To further evaluate the associations of these SNPs with GDM, adjusted logistic regression analysis was also performed by age, pre-pregnancy BMI, family history of diabetes. The results showed that the associations of the five SNPs with GDM were still not significant in all the genotype models (*P >* 0.05).

**Table 2:** Association between VDR gene SNPs and the risk of GDM

	GDM (*n* = 826)	Non-GDM (*n* = 858)	Crude OR (95 % CI)	Crude *P*	Adjusted OR (95 % CI)	Adjusted *P*
**rs2228570**						
Codominant model						
GG	219	230	1(ref.)		1(ref.)	
GA	389	415	0.98(0.78–1.24)	0.89	0.92(0.70–1.21)	0.53
AA	206	203	1.07(0.82–1.39)	0.64	2.40(0.73–1.38)	0.99
Dominant Model						
GG	219	230	1(ref.)		1(ref.)	
GA + AA	595	618	1.01(0.81–1.26)	0.92	0.94(0.73–1.22)	0.66
Recessive Model						
GG + GA	608	645	1(ref.)		1(ref.)	
AA	206	203	1.08(0.86–1.35)	0.52	1.06(0.81–1.38)	0.67
Overdominant model						
GG + AA	425	433	1(ref.)		1(ref.)	
GA	389	415	0.96(0.79–1.16)	0.64	0.92(0.73–1.15)	0.45
**rs1544410**						
Codominant model						
CC	752	776	1(ref.)		1(ref.)	
CT	63	69	0.94(0.66–1.35)	0.74	0.98(0.65–1.49)	0.92
TT	3	3	1.03(0.21–5.13)	0.97	0.64(0.13–3.19)	0.58
Dominant Model						
CC	752	776	1(ref.)		1(ref.)	
CT + TT	66	72	0.95(0.67–1.34)	0.76	0.96(0.64–1.43)	0.82
Recessive Model						
CC + CT	815	845	1(ref.)		1(ref.)	
TT	3	3	1.04(0.21–5.15)	0.97	0.64(0.13–3.20)	0.58
Overdominant model						
CC + TT	755	779	1(ref.)		1(ref.)	
CT	63	69	0.94(0.66–1.35)	0.74	0.98(0.65–1.49)	0.93
**rs739837**						
Codominant model						
GG	414	447	1(ref.)		1(ref.)	
GT	324	327	1.07(0.87–1.31)	0.52	1.10(0.86–1.41)	0.44
TT	78	77	1.09(0.78–1.54)	0.61	1.12(0.80–1.66)	0.56
Dominant Model						
GG	414	447	1(ref.)		1(ref.)	
GT + TT	402	404	1.07(0.89–1.30)	0.46	1.1(0.88–1.39)	0.39
Recessive Model						
GG + GT	738	774	1(ref.)		1(ref.)	
TT	78	77	1.06(0.76–1.48)	0.72	1.08(0.74–1.57)	0.70
Overdominant model						
GG + TT	492	524	1(ref.)		1(ref.)	
GT	324	327	1.06(0.87–1.28)	0.59	1.08(0.85–1.37)	0.52
**rs731236**						
Codominant model						
AA	745	778	1(ref.)		1(ref.)	
GA	68	67	1.06(0.75–1.51)	0.75	1.16(0.77–1.75)	0.48
GG	4	4	1.04(0.26–4.19)	0.95	0.49(0.1–2.21)	0.35
Dominant Model						
AA	745	778	1(ref.)		1(ref.)	
GA + GG	72	71	1.06(0.75–1.49)	0.74	1.10(0.74–1.64)	0.65
Recessive Model						
AA + GA	813	845	1(ref.)		1(ref.)	
GG	4	4	1.04(0. 26-4.17)	0.96	0.48(0.1–2.18)	0.34
Overdominant model						
AA + GG	749	782	1(ref.)		1(ref.)	
GA	68	67	1.06(0.75–1.51)	0.75	1.17(0.77–1.76)	0.47
**rs7975232**						
Codominant model						
CC	415	446	1(ref.)		1(ref.)	
CA	324	328	1.06(0.87–1.30)	0.57	1.10(0.86–1.40)	0.47
AA	77	74	1.12(0.79–1.58)	0.53	1.19(0.79–1.77)	0.40
Dominant Model						
CC	415	446	1(ref.)		1(ref.)	
CA + AA	401	402	1.07(0. 88 − 1.30)	0.48	1.11(0.89–1.40)	0.36
Recessive Model						
CC + CA	739	774	1(ref.)		1(ref.)	
AA	77	74	1.09(0. 78-1.52)	0.61	1.14(0.78–1.68)	0.50
Overdominant model						
CC + AA	492	520	1(ref.)		1(ref.)	
CA	324	328	1.04(0.86–1.27)	0.67	1.07(0.84–1.35)	0.60

Adjusted OR is adjusted for age, pre-pregnancy BMI, family history of diabetes.

Abbreviations: *VDR* vitamin D receptor; *SNPs* single nucleotide polymorphisms; *GDM* gestational diabetes mellitus; *BMI* body mass index; *OR* odds ratio; *CI* confidence interval; *ref* reference genotype

### Gene‐gene interactions to GDM

The analysis of gene-gene interactions indicated that both two-factor model (rs731236, rs7975232) and three-factor model (rs2228570, rs731236, rs7975232) had good cross-validation consistency at 9/10, but the test accuracy of the two-factor model (0.514) was higher than that of the three-factor model (0.511), so the best model was the two-factor gene-gene interaction model. However, as was shown in Table 3, there was no significance of the test set in the two-factor gene-gene interaction (*P* > 0.05). Therefore, it could be speculated that there were no gene-gene interactions on the GDM risk among the five VDR gene SNPs.

**Table 3:** Interaction of two-factor gene-gene model

	Sensitivity	Specificity	*χ*^*2*^	*P*	OR (95 %CI)	Kappa
Training set	0.369	0.669	6.920	0.009	1.323(1.074–1.630)	0.065
Test set	0.377	0.652	0.154	0.695	1.133(0.607–2.116)	0.029
Total set	0.412	0.652	7.202	0.007	1.308(1.075–1.591)	0.063

Abbreviations: *OR* odds ratio; *CI* confidence interval

## Discussion

In the present study, we analyzed the association of VDR gene SNPs rs154410, rs7975232, rs731236, rs2228570 and rs739837 with GDM in Wuhan, China. The results revealed that, VDR gene polymorphic markers were not associated with GDM in central Chinese population. Furthermore, there were no gene-gene interactions on the GDM risk among the examined VDR gene SNPs.

The rs739837 SNP is located at the three-primer untranslated region (3′-UTR) of the VDR gene. This region does not affect amino acid sequence and is not likely to affect the function of the gene [24]. However, the variant rs739837 might affect the expression of VDR gene by binding with microRNA [25]. Several studies investigated the relationship between this locus and T2D and reported that rs739837 was associated with susceptibility to T2D [15, 17, 24]. To date, two studies had studied the relationship between rs739837 and GDM. Shi et al. reported no relationship between the genotypic model of rs739837 and GDM, whereas Wang et al. found a statistical correlation between the rs739837 polymorphism and GDM risk [26, 27]. However, neither of the two studies had analyzed the combined effect with other VDR gene SNPs. As susceptibility was attributable not to a single polymorphism or allele, but rather to multiple polymorphisms [28], we evaluated rs739837 and other four widely studied VDR gene SNPs and their gene-gene interactions on the risk of GDM. The result showed that there was no statistical correlation between rs739837 and GDM. Besides, no evidence was found at the gene-gene interactions. To our knowledge, this study is the first to investigate the combined effect between rs739837 and other VDR gene SNPs on the risk of GDM. The results need to be verified in future studies.

A meta-analysis on rs1544410, rs7975232, and rs731236 with the risk of T2D produced negative results [29]. As for the association between the three polymorphisms and GDM, the results were inconsistent [18, 19, 22, 30]. Our study reported a negative result, which was consistent with the finding of Apaydin et al. [18]. Rs1544410, rs7975232, and rs731236 are all located in the 3′-UTR and have been shown to be in strong linkage disequilibrium [29]. Polymorphisms of rs1544410, rs7975232, and rs731236 are probably non-functional because they are either located in intron (rs1544410 and rs7975232 in intron 8), which will be removed during mRNA post transcriptional modification, or result in no amino acid sequence change (rs731236 in exon 9).

The rs2228570 polymorphism was linked to risk of GDM in Turkish women and Iranian population [18, 31]. However, studies in other countries could not establish association between rs2228570 and GDM [19, 20]. In the present study, we reported no evidence of genotypic association of the rs2228570 polymorphism with GDM in central Chinese population. The rs2228570 is located at the 5′ end region of the VDR gene. It is reported as an independent marker of the VDR gene because it has not been shown to be in linkage disequilibrium with any other VDR polymorphisms [31]. It produces either a 424 or a 427 amino acid VDR protein. These two isoforms are thus structurally distinct, unlike those VDR gene that contain polymorphisms present in the 3′-portion of the gene that are either silent codon changes or are found in introns or in the 3′-untranslated regions [32]. Even the relationship between the rs2228570 polymorphism and T2D is still controversial [14–16, 29], probably because the socio-demographic characteristics, experimental methods, and sample size are different in the studied populations.

Our study had several strengths. First, we employed MDR method to explore the gene-gene interactions on the GDM risk among the selected SNPs. The identification and characterization of gene-gene interactions had been limited mainly by a lack of powerful statistical methods and a lack of large sample size [33]. To overcome these limitations, the MDR method was developed. It was used for detecting and characterizing high-order gene to gene interactions [34] and was shown to have good power in relatively small case-control studies [23, 35]. Second, we adjusted potential confounding factors including age, pre-pregnancy BMI and family history of diabetes to explore the association in different genotype models. At last, we used a relatively large sample size, which was able to provide enough statistical power.

However, there were also some limitations in this study. First, the level of plasma vitamin D was not measured in all subjects and the number of women with previous history of GDM was too small to analyze. We will carry out further study with large sample size to explore the impact of gene polymorphisms on the recurrence of GDM in future. Second, the information of environmental and lifestyle factors was lacked, which had been reported recently to be important determinants of GDM development [36, 37]. Finally, there were some potential biases that came from the cross-sectional nature of the case–control study. Thus, cohort studies concerning the above-mentioned factors will be required in future to validate the findings of the study.

## Conclusions

The VDR gene SNPs rs154410, rs7975232, rs731236, rs2228570 and rs739837 showed non-significant associations with GDM in central Chinese population. Furthermore, there were no gene-gene interactions on the GDM risk among these SNPs.

## Data Availability

The datasets used and analyzed during the current study are available from the corresponding authors on reasonable request.

## Abbreviations

GDM: Gestational diabetes mellitus
T2D: Type 2 diabetes
VDR: Vitamin D receptor
SNPs: Single nucleotide polymorphisms
OGTT: Oral glucose tolerance test
IADPSG: International Association of Diabetes and Pregnancy Study Groups
BMI: Body mass index
MAF: Minor allele frequency
SD: Standard deviation
ANOVA: Analysis of variance
HWE: Hardy-Weinberg Equilibrium
MDR: Multifactor dimensionality reduction
OR: Odds ratio
CI: Confidence interval
3′-UTR: Three-primer untranslated region
